# Somatic Embryogenesis in Olive

**DOI:** 10.3390/plants10030433

**Published:** 2021-02-25

**Authors:** Carolina Sánchez-Romero

**Affiliations:** Departamento de Botánica y Fisiología Vegetal, Universidad de Málaga, Campus de Teatinos s/n, 29071 Málaga, Spain; c.sanchez@uma.es

**Keywords:** biotechnology, breeding, *Olea europaea*, olive, plant regeneration

## Abstract

The olive is a fruit tree species economically very important in countries of the Mediterranean basin. Somatic embryogenesis is a powerful in vitro technique with multiple uses in different fields, including breeding programs performed by both classical and innovative procedures. Thus, somatic embryogenesis enables the application of biotechnological methods such as genetic transformation, somaclonal variation, somatic hybridization, germplasm cryopreservation, in vitro mutagenesis or in vitro selection. This editorial paper presents a special issue focused on “Somatic embryogenesis in olive”. In this manuscript, the conceptual framework of the special issue is established and the contributions are summarized and put into context. Finally, the main bottlenecks limiting the practical applicability of somatic embryogenesis in this species are identified and the future research prospects are discussed.

## 1. Introduction

The olive (*Olea europaea* L.) is one of the most important and widespread fruit trees in the Mediterranean basin [1]. This evergreen, long-lived tree is cultivated for its fruits and oil, which constitutes an important component of the Mediterranean diet [2]. High nutritional quality and healthy effects of olive oil compared with other plant oils greatly increased worldwide olive oil consumption in the last years [3,4], thus raising its commercial value [2] and economic importance.

Although olive has been cultivated in the Mediterranean basin from ancient times [5], increased interest in olive products has led to worldwide expansion of olive tree plantations. New countries such as Argentina, Chile, Mexico, USA, Japan, China, New Zealand, Australia, and South Africa have introduced its cultivation in the last years [6,7] and the cultivated area has increased from 8,351,779 ha in 2000 to 10,578,246 ha in 2019 [8], thus making olive one of the most extensively cultivated fruit crops in the world [9]. Nevertheless, about 90% of olive groves still concentrates in the Mediterranean basin [8], with Spain, Italy, and Morocco accounting for over 51% of the 19,464,495 tons of olives worldwide produced [8].

Somatic embryogenesis is the developmental process through which a somatic cell or group of somatic cells give rise to an embryo, capable of developing into a whole plant [10]. Somatic embryogenesis can be initiated through several pathways: (1) Direct embryogenesis from single cells through a totipotent zygotic-like state, (2) direct embryogenesis dependent on seed/embryo identity factors, and (3) indirect embryogenesis from embryogenic cell clusters [11]. The switch from a somatic to an embryogenic state implies coordinated changes at multiple levels produced by exogenous plant growth regulators or stress treatments [12,13]. Subsequently, plant growth regulators, among other physical and chemical treatments, regulate the transition between each embryonic developmental stage up to embryo conversion into plants [14].

Somatic embryogenesis presents multiple applications, such as: (1) Utilization as a model system in embryological studies and investigations on the cellular, molecular, and genetic mechanisms involved in the acquisition of competence for somatic embryogenesis [15,16] or cell differentiation [13,17]. (2) Large-scale propagation of selected genotypes. (3) Production of synthetic seeds. (4) Germplasm conservation. (5) Use as adventitious regeneration method, thus allowing the exploitation of biotechnological techniques such as genetic transformation, somaclonal variation, in vitro mutagenesis, production of haploid and double-haploid plants, somatic hybridization, and in vitro selection against biotic or abiotic stressing agents [10]. Therefore, somatic embryogenesis is a powerful in vitro technique, with multiple possibilities of use in olive-breeding programs based in both conventional and innovative methods.

## 2. Somatic Embryogenesis in Olive

The olive is a tissue culture recalcitrant species [18] and, as in other trees, somatic embryogenesis is the main method for adventitious plant regeneration [19,20].

Somatic embryogenesis in olive was first reported by Rugini [21], who initiated embryogenic cultures from immature zygotic embryos of the cultivars Dolce Agogia, Leccino, Frantoio, and Moraiolo. Since then, olive embryogenic cultures have been induced from juvenile tissues of multiple cultivars and, in scarce occasions, from explants of adult origin [20]. Somatic embryogenesis protocols currently available allow the obtainment of an acceptable number of plants from embryogenic cultures of juvenile origin [22,23].

Application of biotechnological tools to olive through somatic embryogenesis-based systems has greatly progressed in the last years [20]. Genetic transformation is the innovative technique most widely applied, having been used with different aims, such as, growth habit modification [24], drought [25] and osmotic stress tolerance [26], or pathogen resistance [27,28]. Somaclonal variation occurrence has been investigated in olive embryogenic cultures and different somaclones with altered vegetative and reproductive characters have been identified [29,30,31]. Cryopreservation of different embryogenic tissues has been performed using both slow cooling and vitrification-based methods [32,33,34,35,36]. The droplet vitrification method in aluminum foil strips gave rise to very good results allowing the safe long-term storage of both embryogenic calli and somatic embryos [32,36].

This special issue includes four articles on somatic embryogenesis in olive. The first two works address the optimization of somatic embryogenesis in specific genotypes, while the last two manuscripts focus on the effect of cryopreservation on the somatic embryogenesis process and the quality of the regenerated plants. This is an important issue deserving attention as some of the somatic embryogenesis applications involve its execution in combination with other biotechnological methods.

Pires et al. [37] developed a somatic embryogenesis protocol for the Portuguese cultivar Galega vulgar. The embryogenic competence of different explants excised from mature zygotic embryos was investigated following a two-step procedure. The best results were attained from radicles cultured under a 16 h photoperiod. Embryogenic cultures were maintained in ECO medium [38,39] under light conditions and embryo conversion was achieved in plant growth regulator free-OMc medium, not being necessary very rigorous maturation requirements. Regenerated plants successfully acclimatized to ex vitro conditions. This is the first investigation reporting somatic embryogenesis from embryo-derived explants of the cultivar Galega vulgar.

Mazri et al. [40] optimized the maturation and conversion of somatic embryos derived from radicle segments of mature zygotic embryos of the cultivar Dahbia. Different culture media and plant growth regulator treatments were tested to induce the maturation of globular embryos selected from maintenance medium. Similarly, cotyledonary embryos developed in maturation medium were subjected to different culture media, hormone combinations and light conditions in order to induce germination. Sucrose and mannitol pretreatments were also applied to matured embryos, although no improvement in embryo conversion was obtained. Secondary embryogenesis and adventitious bud formation were observed in non-germinating somatic embryos. The frequency of these morphogenic responses depended on culture conditions.

Bradaï and Sánchez-Romero [41] evaluated the effect of cryopreservation on the different phases of the somatic embryogenesis process. For this purpose, the behavior of cultures established from cryopreserved somatic embryos was compared with that of control, non-cryopreserved cultures during the proliferation, maturation, and germination steps. The results obtained revealed that the droplet-vitrification method optimized by Bradaï et al. [32] for the cryopreservation of olive somatic embryos did not negatively affect somatic embryogenesis executed following the protocol described by Sánchez-Romero [23]. The genotype played a key role, largely determining the effect of cryopreservation on the different phases of somatic embryogenesis.

In a second article, Bradaï and Sánchez-Romero [42] investigated the influence of cryopreservation on the regeneration performance of olive embryogenic cultures and the quality of the plants obtained, analyzing their behavior on the subsequent steps required for ex vitro plant establishment. No effect of cryopreservation could be observed on the regeneration potential or the regenerated plants. No influence on shoot multiplication, rooting and acclimatization was detected either, although a significant genotype × cryopreservation interaction was found for shoot length during the multiplication step.

## 3. Conclusions and Future Prospects

Although much progress has been accomplished in olive somatic embryogenesis in the last years [20], some drawbacks still limit its practical applicability in this species.

Though different somatic embryogenesis protocols are available in olive [20], their efficiency varies depending on the genotype [20,22,41]. This influence of the genetic constitution has been observed in the different phases of this multi-step technique, from the initiation of embryogenic cultures [21,43] to the germination of somatic embryos [40]. This high genotype dependence limits the applicability of the established procedures, hindering protocols standardization.

Basic studies carried out at multiple levels (molecular, cellular, physiological, genetic, or epigenetic) on key events of the somatic embryogenesis process, such as dedifferentiation, acquisition of the embryogenic competence, or the transition thorough the successive embryonic developmental stages can contribute to elucidate the mechanisms underlying somatic embryogenesis. These investigations can help to understand the effects of the treatments tested, thus improving the efficiency of protocol optimization and minimizing trial and error experimentation. Additionally, knowing factors regulating key processes or shifts in this morphogenic program may contribute to the development of more general, widely applicable procedures.

Although limitations at different steps of the somatic embryogenesis process have been reported in olive [44], induction of embryogenic cultures from adult tissues can be nowadays considered the most limiting step. As only the initiation from explants of adult origin can ensure genetic conformity, this point must be regarded as a fundamental aspect determining the applicability of somatic embryogenesis. Although Rugini and Caricato [45] and Capelo et al. [46] reported successful somatic embryogenesis initiation from adult material of the cultivars Canino and Moraiolo and one genotype of wild olive, respectively, the protocols utilized were not applicable to other genotypes. More recently, Mazri et al. [47] developed a protocol for the induction of somatic embryogenesis from petioles and leaf fragments of plants of the cultivar Dahbia rejuvenated by repetitive micropropagation. This procedure has been successfully reproduced in two genotypes of wild olive, using apical buds excised from micropropagated shoots [48]. Toukif et al. [49] also obtained somatic embryogenesis from leaves and petioles derived from micropropagated plants of the cultivar Picual. Although the induction medium contained the hormonal combination previously used by Mazri et al. [47], significantly longer induction periods were tested and the expression of somatic embryogenesis was performed in a plant growth regulator-free medium.

An important aspect in somatic embryogenesis is the fidelity of the regenerated plants. As in other in vitro culture techniques, somaclonal variation is one of the more important drawbacks of somatic embryogenesis. Somaclonal variation, defined by Larkin and Scowcroft [50] as the variation arising in the cell cultures, regenerated plants, and their progenies as a consequence of the plant cell culture itself, can have multiple origins, such as chromosomal aberrations, genetic alterations, or epigenetic changes [51]. According to Hervé et al. [51], these different defects are not mutually exclusive, and several different sources of somaclonal variation can be observed within the same regenerant population. 

In olive plants regenerated via somatic embryogenesis, both true-to-typeness and somaclonal variation have been reported. Thus, Rugini and Silvestri [44] found phenotypic stability in in-field observations of plants of the cultivar Canino derived from somatic embryogenesis. However, in plants of the cultivar Frangivento, Leva et al. [31] described two somaclones exhibiting altered vegetative and reproductive traits. In plants regenerated from zygotic embryos of the cultivar Picual, somaclonal variation was detected by both phenotypic [29] and genetic analysis [30]. Phenotypic analysis allowed the identification of fourteen variant phenotypes affecting vegetative and reproductive development. Some of the identified variants can constitute a very interesting material to be included in olive breeding programs [29].

Therefore, although much more progress has been achieved in olive in the last years, some drawbacks still limit its practical applicability supporting breeding programs based in both conventional and innovative techniques. Until such weak points are solved, more research is still needed. 
